# Applied high-intensity interval cardio yoga improves cardiometabolic fitness, energetic contributions, and metabolic flexibility in healthy adults

**DOI:** 10.3389/fphys.2023.1279505

**Published:** 2023-10-17

**Authors:** So-Young Park, Woo-Hwi Yang

**Affiliations:** ^1^ Graduate School of Sports Medicine, CHA University, Pocheon-si, Gyeonggi-do, Republic of Korea; ^2^ Department of Medicine, General Graduate School, CHA University, Pocheon-si, Gyeonggi-do, Republic of Korea

**Keywords:** metabolism, physiological adaptation, lactate, aerobic capacity, V˙O_2max_

## Abstract

**Purpose:** Currently, there is no interventional approach to increase the intensity of Surya Namaskar a popular hatha yoga sequence used worldwide. Therefore, this study investigated how tempo-based high-intensity interval cardio yoga (HIICY) and traditional interval hatha yoga (TIHY) affects cardiometabolic fitness in active adults.

**Methods:** Twenty physically active male and female individuals were randomly separated into HIICY (5 males, 5 females, 1.5 s tempo) and TIHY (5 males, 5 females, 3 s tempo) groups. The intervention included twelve exercise sessions for 4 weeks in both groups. Participants conducted a ramp test to determine their maximal oxygen uptake ( $\dot{V}$ O_2max_), maximal velocity at $\dot{V}$ O_2max_ (v $\dot{V}$ O_2max_), and maximal heart rate (HR_max_). Afterward, they performed a 10-min high-intensity cardio yoga test (HICYT) to determine heart rate (HR_peak_ and HR_mean_), oxygen uptake ( $\dot{V}$ O_2peak_ and $\dot{V}$ O_2mean_), respiratory exchange ratio (RER), blood lactate concentrations (La^−^_peak_ and ∆La^−^), fat and carbohydrate oxidations (FATox, CHOox), and energetic contributions (oxidative; *W*_Oxi_, glycolytic; *W*_Gly_, and phosphagen; *W*_PCr_, total energy demand; *W*_Total_).

**Results:** $\dot{V}$ O_2max_ and v $\dot{V}$ O_2max_ showed time and group × time interactions (*p* < 0.01, *p* < 0.0001, *p* < 0.001, respectively). $\dot{V}$ O_2max_ after HIICY was significantly higher than in pre-testing and following TIHY (*p* < 0.001, *p* < 0.0001, respectively). $\dot{V}$ O_2peak_, $\dot{V}$ O_2mean_, RER, HR_peak_, and HR_mean_ during the 10-min HICYT showed significant time effects (*p* < 0.05). ∆La^−^ indicated a group × time interaction (*p* < 0.05). Group x time interaction effects for FATox at the fourth and sixth minute were observed (*p* < 0.05, respectively). Absolute (kJ) and relative (%) *W*_Oxi_, *W*_Gly_, and *W*_Total_ showed time and group × time interaction effects (*p* < 0.05, *p* < 0.01, respectively). Furthermore, %*W*_Gly_ was reduced following HIICY (*p* < 0.05). Additionally, $\dot{V}$ O_2max_ and v $\dot{V}$ O_2max_ were highly correlated with *W*_Oxi_ in kJ (*r* = 0.91, 0.80, respectively). Moderate to high correlations were observed among CHOox, FATox, and absolute $\dot{V}$ O_2max_ (*r* = 0.76, 0.62, respectively).

**Conclusion:** A 4-week period of HIICY improved cardiometabolic fitness, oxidative capacity, and metabolic flexibility compared with TIHY, in physically active adults. Therefore, HIICY is suitable as HY-specific HIIT and time-efficient approach for relatively healthy individuals.

## Introduction

The World Health Organization’s (WHO) definition of “physical inactivity” refers to the failure to meet recommended levels of weekly physical activity. This guideline consists of at least 150 min of moderate-intensity or 75 min of high-intensity aerobic activity per week (Santos et al., 2023). It is now well appreciated that physical inactivity is the fourth leading cause of death and has been reported to contribute to multiple chronic diseases including insulin resistance, type 2 diabetes mellitus, obesity, metabolic and endocrine diseases (Balakumar et al., 2016; Kerr and Booth, 2022). The global economic burden of non-communicable diseases associated with lack of physical activity was estimated to be between US$ 53.8 and 67.5 billion as of 2016 (Scheltens et al., 2021; Santos et al., 2023). One increased metabolic equivalent of task (1 MET = $\dot{V}$ O_2_ 3.5 mL·kg^−1^·min^−1^) at the maximal aerobic capacity was related to 13% and 15% risk reductions for all-cause mortality and coronary heart/cardiovascular disease respectively in males and females (Kodama et al., 2009). Furthermore, the prevalence of metabolic diseases was reduced by 6.3-fold in males and 4.9-fold in females.

Cardiorespiratory fitness consists of a wide range of parameters between an aerobic base and maximal aerobic exercise capacity, which represents maximal aerobic performance and the functional capacity of numerous bodily systems. These can be determined by maximal oxygen uptake ( $\dot{V}$ O_2max_) (Kodama et al., 2009; Meyler et al., 2021; Yang et al., 2022a; Hwang et al., 2022). $\dot{V}$ O_2max_ is a crucial indicator for maximal aerobic performance and cardiorespiratory function in different individuals and athletes, and also provides detailed insights into inter-individual prescriptions in exercise physiology and sports science (Bosquet et al., 2002; Wisløff et al., 2007; Niemeyer et al., 2021). Moreover, WHO considers $\dot{V}$ O_2max_ as one of the valuable indicators or markers for cardiorespiratory fitness or health, and it is strongly associated with better physical performance. Epidemiological data from previous studies revealed that having a proportionally high $\dot{V}$ O_2max_ is a potent sign of health and life expectancy in all age groups (Ross et al., 2016; Udhan et al., 2018). Furthermore, exercise-induced acute cardiorespiratory adaptations enhance the ability of the cardiovascular system to meet the demands of skeletal muscle exercise, via increases in pulmonary ventilation, heart rate, stroke volume, and cardiac output with moderate increases in systolic blood pressure, peripheral vasoconstriction, and vasodilation (Weiner and Baggish, 2012; Predel, 2014).

In terms of improving $\dot{V}$ O_2max_, the most effective intervention is high-intensity interval training (HIIT) (Buchheit and Laursen, 2013a; Batacan et al., 2017; MacInnis and Gibala, 2017; Sabag et al., 2022; Jacob et al., 2023). It is well known that HIIT protocols can maximally induce the oxygen uptake and utilization system for more than 85% of $\dot{V}$ O_2max_ or peak oxygen uptake ( $\dot{V}$ O_2peak_) and thus provide the most effective stimulation to increase $\dot{V}$ O_2max_ (Buchheit and Laursen, 2013a; Buchheit and Laursen, 2013b; Sheykhlouvand et al., 2018).

HIIT prescription is used as an alternative physical exercise for those who do not have enough time contemporary people and is also consistently ranked in the top 10 fitness trends of the American College of Sports Medicine (ACSM) (Sabag et al., 2022; Kercher et al., 2023). Previous studies have reported that HIIT is efficient at developing the three energy systems utilized in humans in a time-efficient manner, which includes active or passive recovery following repeated rounds of exercise. HIIT protocols are designed to reach an exercise intensity such as >90% of maximal heart rate (HR_max_), the second ventilatory threshold (>VT_2_), over the second lactate threshold (>4 mmol·L^-1^; zone 3: high-intensity exercise), and >85% of $\dot{V}$ O_2max_ and $\dot{V}$ O_2peak_ (Jamnick et al., 2020; Protzen et al., 2020).

In terms of metabolism, metabolic flexibility is defined as the ability to rapidly convert to generate adenosine triphosphate (ATP) from efficient fat and carbohydrate utilization based on physiological demands (Goodpaster and Sparks, 2017; Galgani et al., 2022). Efficient metabolic flexibility is essential to prevent metabolic diseases directly related to physical inactivity-induced mitochondrial dysfunction (San-Millán and Brooks, 2018; Yang et al., 2022a). HIIT increases metabolic flexibility, which indicates a strong relationship with metabolic health parameters such as insulin sensitivity and mitochondrial respiratory capacity (Aparecido et al., 2022).

In this regard, yoga is a mind-body practice consisting of physical postures (Asana), breathing techniques (Pranayama), and meditation (Dhyana) (Patwardhan, 2017). Notably, yoga is one of the most practiced complementary or alternative exercise interventions to achieve optimal physical and mental health (Udhan et al., 2018; Khoshnaw and Ghadge, 2021). Furthermore, hatha yoga (HY) is becoming increasingly popular in the United States and Europe as an alternative form of physical activity that can support individuals to reach globally recommended levels of physical activity (Larson-Meyer, 2016). The main goals of HY are to enable practitioners to improve body, breath, and spirit states and to “prepare a healthy mind and body to immerse oneself in meditation for self-realization” (Schmalzl et al., 2015). A specific set of 19 asanas called Surya Namaskar B (Sun Salutations, SS) includes the vinyasa system and is one of the most basic and representative sequences in many styles of yoga classes worldwide (Papp et al., 2016; Brinsley et al., 2022).

The average metabolic cost during HY focusing on various postures, alignments, and breathing for different times is less than 3 METs and show results similar to walking on a treadmill at 3.2 km·h^−1^ (Hagins et al., 2007; Larson-Meyer, 2016). However, HY (Surya Namaskar) lasts at least 10 min and can contribute sufficiently intense physical activity to improve an individual’s cardiorespiratory fitness with 4-6 METs, corresponding to ACSM’s moderate-to-high-intensity physical activity guidelines (Hagins et al., 2007; Mody, 2011; Larson-Meyer, 2016; Potiaumpai et al., 2016). A recent study by Lee et al. (2021) reported that HY, which consisted of 1.5 s for each asana and lasted for 10 min, could be used as HIIT in physically active individuals. The results of this study showed %HR_peak_ of HR_max_, %HR_mean_ of HR_max_, METs of $\dot{V}$ O_2peak_ and $\dot{V}$ O_2mean_, and blood lactate concentrations (La^−^) values during high-intensity HY (HIHY), that reached 95.6%, 88.7%, 10.54, and 8.67 METs, and 8.31 mmol·L^−1^ La^−^, respectively. This HIHY indicated suitable levels for HIIT. However, no interventional approach to HIHY was performed in this study. It was unclear therefore, whether high-intensity interval cardio yoga (HIICY) as HIHY could improve $\dot{V}$ O_2max_, energetic contributions, and metabolic flexibility in physically active individuals.

Therefore, the aim of the study was to investigate how at least 4-week tempo-based HIICY and traditional interval hatha yoga (TIHY) practices affected cardiometabolic fitness parameters, such as $\dot{V}$ O_2max_, energetic contributions, and metabolic flexibility.

## Materials and methods

### Participants

**FIGURE 1:**
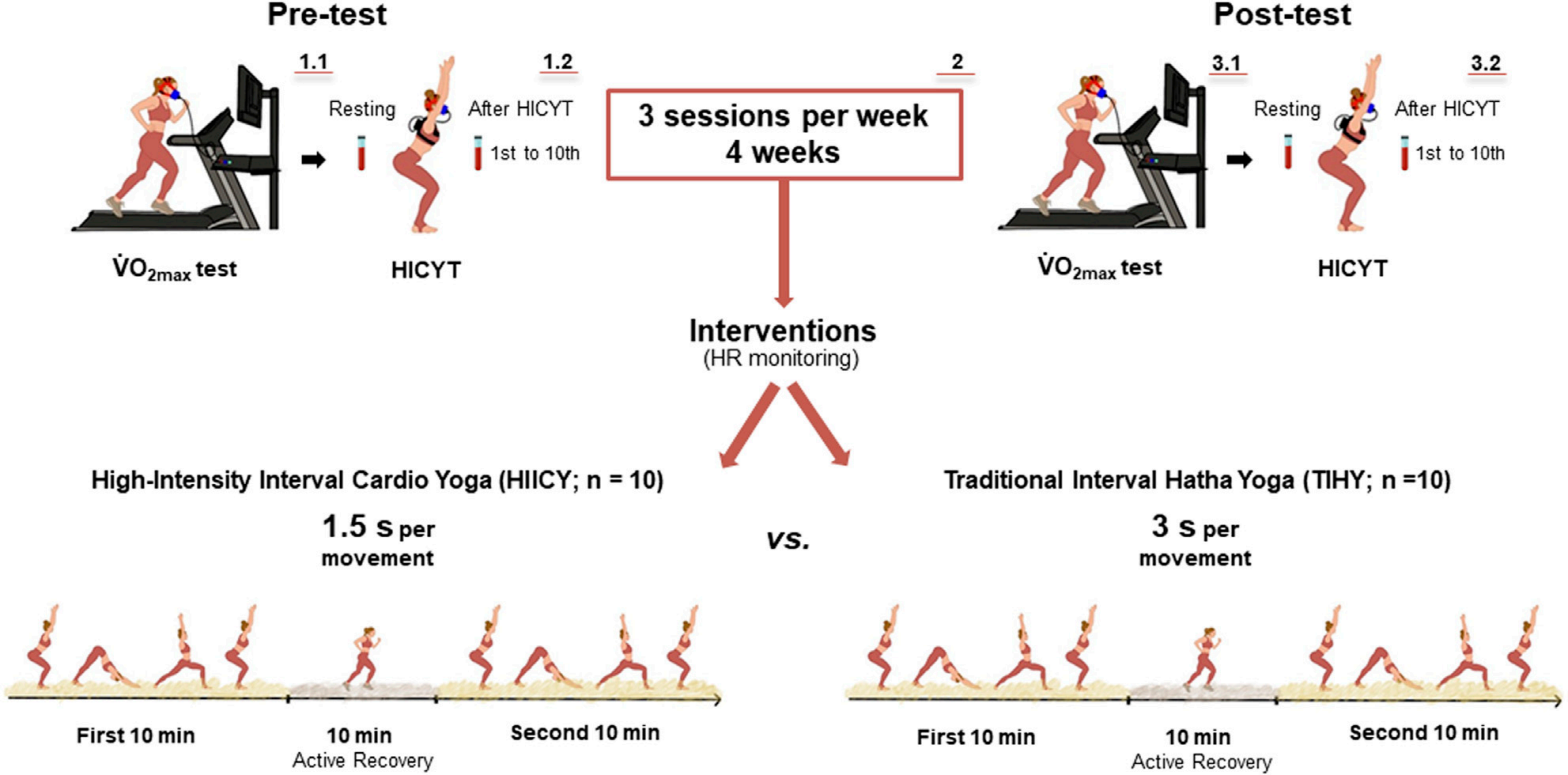
Study procedure. The pre-test including $\dot{V}$ O_2max_ (1.1) and HICYT (1.2) was conducted before the 4-week HIICY and TIHY training (2) commenced. The same set-up was utilized for post-testing (3.1 and 3.2). All participants conducted the HICYT (n = 20) after the $\dot{V}$ O_2max_ test and were randomly separated into HIICY (1.5 s tempo, n = 10, males 5 and females 5) and TIHY (3 s tempo, n = 10, males 5 and females 5) groups. Capillary blood was collected from the earlobe (20 μL) before and at 1-min intervals (1st to 10th) after the 10-min HICYT. $\dot{V}$ O_2max_; maximal oxygen uptake, HICYT; high-intensity cardio yoga test, HIICY; high-intensity interval cardio yoga, TIHY; traditional interval hatha yoga. The numbers indicate the flow of the test procedures.

The sample size for the study was calculated using G*Power software version 3.1.9.4 (Heinlich Heine University, Düsseldorf, Germany) which considered: effect size = 0.3, alpha error probability = 0.05, and statistical power = 0.8. The effect size was determined based on previous studies (Astorino et al., 2012; Ormsbee et al., 2015; Lee et al., 2021). The total required sample size was calculated to be twenty participants assuming a 10% dropout rate (n = 20). Participants were randomly separated into two tempo-based HY groups: ten physically active individuals were assigned high-intensity interval cardio yoga (1.5 s tempo HIICY; five males and five females) and another 10 participated in traditional interval hatha yoga (3 s tempo TIHY; five males and five females) (Figure 1). All participants satisfied the minimum physical activity standards per week based on WHO guidelines and had no pre-existing cardiovascular, pulmonary, or metabolic diseases or musculoskeletal disorders (Santos et al., 2023). All participants were yoga beginners. Therefore, before the experiment began, they learned the movement sequences with the yoga instructor and quickly became familiar with the movements and sequences through the qualified yoga instructor’s demonstrations during the intervention. The participants maintained their previous physical activity levels throughout the 4-week intervention period and performed no additional training. Anthropometric measurements of all participants were taken in the fasting state. The data were as follows (mean ± standard deviation; SD): age 30 ± 5 years, height 169.6 ± 7.4 cm, body mass 66.8 ± 12.8, body fat 23.4% ± 7.5%, and BMI of 23.0 ± 3.3 kg·m^−2^. The anthropometric data of HIICY and TIHY groups were not significantly different (Table 1). Participants were instructed not to change their diet during the intervention and did not take any medication before and during pre- and post-tests and abstained from nicotine and alcohol for 24 h prior to the testing. This study was approved by the Institutional Review Board of CHA University (IRB No. 1044308-202206-HR-031-02). The approved protocols followed the ethical standards in the Declaration of Helsinki. All participants signed informed consent forms.

**TABLE 1:** Anthropometric data.

Parameters	HIICY (n = 10) (Mean ± SD)	TIHY (n = 10) (Mean ± SD)	All participants (n = 20) (Mean ± SD)
Age (years)	27.6 ± 4.4	32.7 ± 5.2	30.2 ± 5.3
Height (cm)	169.7 ± 7.7	169.5 ± 7.5	169.6 ± 7.4
Body weight (kg)	66.2 ± 12.9	67.3 ± 13.4	66.8 ± 12.8
Body fat (%)	24.5 ± 7.3	22.3 ± 8.0	23.4 ± 7.5
BMI (kg·m^−2^)	22.9 ± 3.6	23.2 ± 3.2	23.0 ± 3.3

There was no significant difference of anthropometric data between HIICY and TIHY groups. HIICY; high-intensity interval cardio yoga, TIHY; traditional interval hatha yoga. ns; *p >* 0.05.

### Study procedure

All participants attended two laboratory visits for pre- and post-testing between 4 weeks. Testing was performed at a temperature of 23°C and relative humidity of 50%. Anthropometric data of participants were analyzed using an eight-electrode segmental multi-frequency bioelectrical impedance analyzer (BIA: 20–100 kHz; InBody 270; InBody Co. Ltd., Seoul, Republic of Korea). All participants rested for 2 h after lunchtime and conducted a ramp test for $\dot{V}$ O_2max_ on a treadmill (NR30XA, DRAX Corporation Ltd., Seoul, Republic of Korea). Afterward, a high-intensity cardio yoga test (HICYT) (one set) was performed on the same testing day. $\dot{V}$ O_2max_ and HICYT post-testing were conducted the same as pre-testing (Figure 1).

### Maximal oxygen uptake ( $\dot{V}$ O_2max_) test

The pre- and post-ramp tests were performed via continuous incremental ramp protocols on a treadmill with the breath-by-breath method using a portable gas analyzer (MetaMax 3B; Cortex Biophysik, Leipzig, Germany). The gas analyzer was calibrated before each test with 15% O_2_ and 5% CO_2_ (Cortex Biophysik, Leipzig, Germany) and the turbine volume transducer was calibrated using a 3-L syringe (Hans Rudolph, Kansas, United States). An initial warm-up was conducted for 10 min by running at 70% of the estimated HR_max_ (Helgerud et al., 2022). The ramp protocol was applied for $\dot{V}$ O_2max_ determination based on a previous study (Sperlich et al., 2015). The initial running speed was 9.0 km·h^−1^ at 2% inclination in 2 min. Then, the running speed was increased by 0.72 km·h^−1^ every 30 s. The investigator verbally encouraged participants to maintain effort for as long as possible to evaluate their maximum aerobic performance seen as reaching a “plateau”. The test was stopped when $\dot{V}$ O_2_ plateau and respiratory exchange ratio (RER) >1.0 were reached, or until volitional exhaustion by the participant (Jurov et al., 2023; Wiecha et al., 2023). The plateau was determined using the method (<2 mL·kg^−1^·min^−1^) that has been explained in previous studies (Krustrup et al., 2005; Niemeyer et al., 2021). Furthermore, $\dot{V}$ O_2max_ was determined as an averaged value of oxygen uptake during the 15-s duration at the end of the plateau (Midgley et al., 2007; Niemeyer et al., 2021; Wiecha et al., 2023). The HR data were recorded using a Polar H10 sensor (Polar Electro, Kemple, Finland). HR_max_ was determined as values through the same section at $\dot{V}$ O_2max_ (Krustrup et al., 2005). Finally, the absolute and relative $\dot{V}$ O_2max_, and velocity at $\dot{V}$ O_2max_ (v $\dot{V}$ O_2max_) were determined (Billat et al., 1996; Riboli et al., 2022).

### High-intensity cardio yoga test (HICYT) and 4-week HIICY and TIHY interventions

**FIGURE 2:**
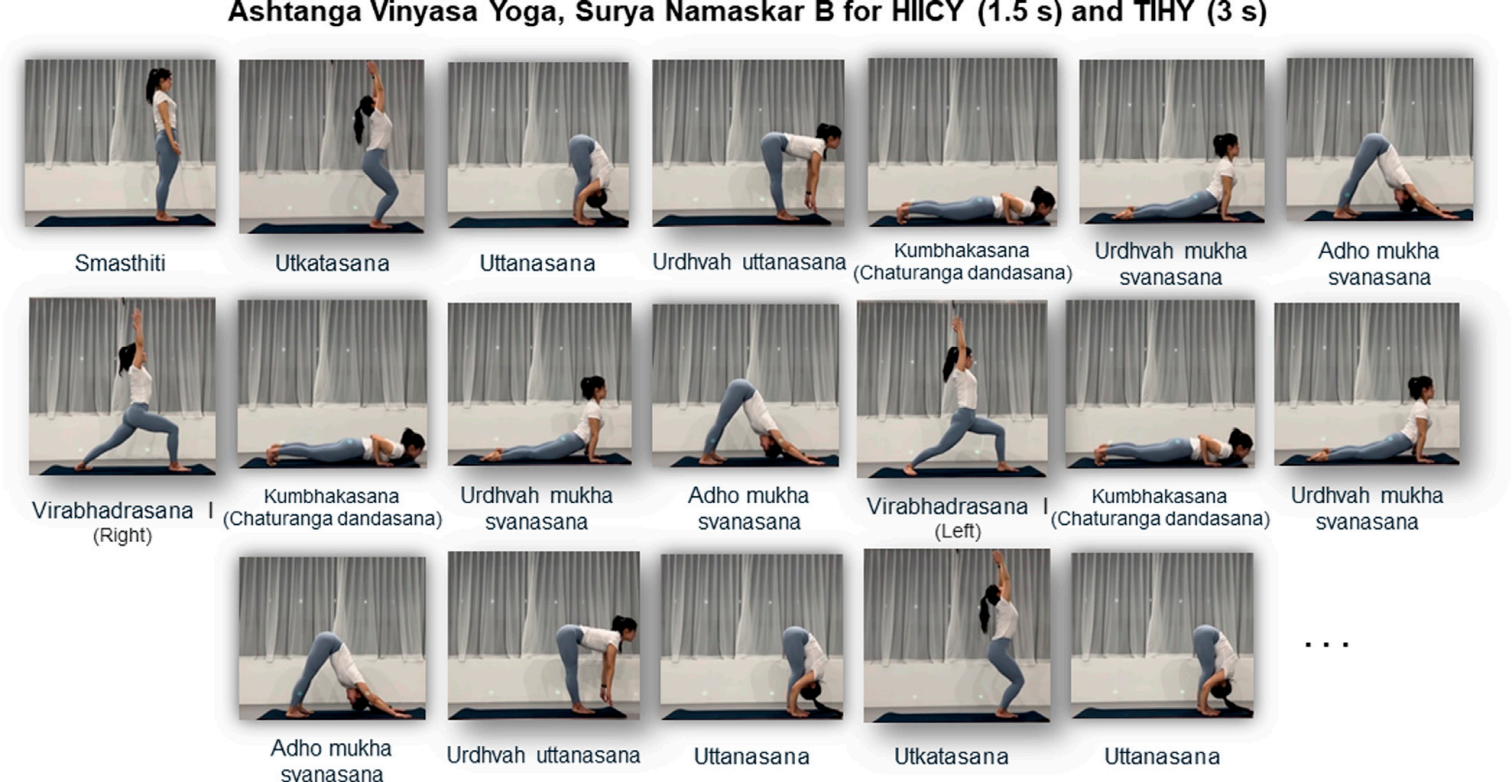
Ten-minute Surya Namaskar B sequence. Interval cardio yoga consists of 10-min of active recovery by walking between two sets of 10-min tempo-based Surya Namaskar B; the total duration of HY intervention was 30 min. HIICY; high-intensity interval cardio yoga, TIHY; traditional interval hatha yoga.

After $\dot{V}$ O_2max_ testing, the participants were advised to continue low-intensity jogging until blood lactate levels were below 2.0 mmol·L^−1^ (Schünemann et al., 2023). They wore a portable gas analyzer and performed a high-intensity cardio yoga test (HICYT) (one set) for 10-min to analyze highest oxygen uptake ( $\dot{V}$ O_2peak_), metabolic flexibility (fat and carbohydrate oxidation; FATox and CHOox) and contributions from the three-energy system. Upon completion of the pre-test, all participants were randomly assigned to either HIICY or TIHY groups. They performed three interventional HIICY and TIHY sessions per week over 4 weeks (a total of 12 sessions) (Costigan et al., 2015; Schmitz et al., 2018) (Figure 1). All training sessions were performed under the supervision of a qualified yoga instructor. For both training sessions and HICYT sequences, the Surya Namaskar B sequence was used which consisted of 19 SS physical exercises (Asanas) (Figure 2). Tempo-based, each movement lasted 1.5 s for HIICY and 3 s for TIHY using a metronome, respectively (Lee et al., 2021). The entire duration for both styles was 30 min, which consisted of 2 sets of 10-min (Sandbakk et al., 2013) HIICY or TIHY and 10-min active recovery between sets. Active recovery was conducted by walking at 40%–45% of the estimated HR_max_, as suggested by a previous study (Lee et al., 2021). The post-test was performed within 2 days of the last training session. Furthermore, HR levels were recorded during the 4-week interventions, and were digitally saved by the HR application for monitoring each training session (Table 2).

**TABLE 2:** HR_mean_ data of 4-week HIICY and TIHY interventions (HIICY; n = 10 and TIHY; n = 10).

Group	HR_mean_ (beats·min^-1^) (Mean ± SD)	% of HR_max_ (mean ± SD)
HIICY	178 ± 3.7	92.7 ± 2.5
TIHY	128 ± 16.3	67.2 ± 8.3

HR_mean_, mean heart rate; % of HR_max_, estimated maximal heart rate percentages; HIICY, high-intensity interval cardio yoga; TIHY, traditional interval hatha yoga.

### Calculations of fat and carbohydrate oxidation rate during HICYT

During HICYT, $\dot{V}$ O_2_ and carbon dioxide ( $\dot{V}$ CO_2_) production were used to calculate metabolic flexibility in terms of fat (FATox) and carbohydrate (CHOox) oxidation, using stoichiometric equations according to previous studies (Jeukendrup and Wallis, 2005; San-Millán and Brooks, 2018; Yang et al., 2022a).

$$\text{FATox }\left(g∙\min^{-1}\right)=1.67∙\dot{V}O_{2}\;\left(L∙\min^{-1}\right)–\;1.67∙\dot{V}{\text{CO}}_{2}\;\left(L∙\min^{-1}\right)$$

$$\text{CHOox }\left(g∙\min^{-1}\right)=4.55∙\dot{V}{\text{CO}}_{2}\;\left(L∙\min^{-1}\right)–\;3.21∙\dot{V}O_{2}\;\left(L∙\min^{-1}\right)$$

### Calculations of energy system contributions (PCr-La^−^-O_2_ method) during HICYT

The contributions of three energy systems (phosphagen [*W*_PCr_], glycolytic [*W*_Gly_], and oxidative [*W*_Oxi_] in kJ and %) were calculated by the PCr-La^−^-O_2_ method (Julio et al., 2017; Yang et al., 2018; Yang et al., 2022b; Kaufmann et al., 2022; Yang et al., 2023). Oxygen consumption parameters (resting oxygen consumption, $\dot{V}$ O_2rest_; average oxygen consumption during the 10-min HICYT, $\dot{V}$ O_2mean_; highest oxygen consumption during the 10-min HICYT, $\dot{V}$ O_2peak_; and fast component of excess oxygen consumption [EPOC_fast_]; and *off* $\dot{V}$ O_2_ kinetics) were assessed at 5-min rest, during, and after 10-min HICYT (6 min), by the breath-by-breath method using a mobile gas analyzer. $\dot{V}$ O_2mean_ and $\dot{V}$ O_2peak_ were determined as the average and highest values during the 10-min HICYT, respectively. Capillary blood was collected from the earlobe (20 μL) before, and at 1-min intervals (1st to 10th) after 10-min HICYT to determine resting and maximal blood lactate concentrations (La^−^_rest_ and La^−^_peak_; the peak value of La^−^ among 10 values) using an enzymatic-amperometric sensor chip system (Biosen C-line; EKF diagnostics sales, GmbH, Barleben, Germany).

The *W*_Oxi_ was calculated by subtracting $\dot{V}$ O_2rest_ from $\dot{V}$ O_2_ by the trapezoidal method in which the domain under the O_2_ data was divided into sections and the summarized trapezoid was utilized to estimate the integral (de Campos Mello et al., 2009; Yang et al., 2022b; Yang et al., 2023). $\dot{V}$ O_2rest_ was determined in a standing position on the yoga mat with the last 30 s of a 5 min period applied as a reference (di Prampero and Ferretti, 1999; Beneke et al., 2004; Julio et al., 2017; Yang et al., 2018; Kaufmann et al., 2022; Yang et al., 2022b).

The *W*_Gly_ analysis was performed by La^−^_rest_ and La^−^_peak_ values, assuming that an accumulation of 1 mmol·L^−1^ was equivalent to 3 mL O_2_·kg^−1^ of body mass (di Prampero and Ferretti, 1999). Delta La^−^ (∆La^−^) was calculated as the difference between La^−^_peak_ after 10-min HICYT and La^−^_rest_ before (Beneke et al., 2004; Campos et al., 2012; Yang et al., 2018; Yang et al., 2022b).

The *W*_PCr_ value was calculated using $\dot{V}$ O_2_ after 10-min HICYT and the fast component of excess post-exercise (Gastin, 2001; Beneke et al., 2004; Yang et al., 2022b; Yang et al., 2023). *Off* $\dot{V}$ O_2_ kinetics were fitted by mono-exponential and bi-exponential models using OriginPro 2021 statistical software (OriginLab Corp, Northampton, USA). The slow component of the bi-exponential model was negligible. Thus, $\dot{V}$ O_2_ values after 10-min HICYT were fitted using a mono-exponential model and *W*_PCr_ was estimated by calculating the integral of the exponential domain (Beneke et al., 2004; Campos et al., 2012; Julio et al., 2017; Yang et al., 2018; Yang et al., 2022b; Kaufmann et al., 2022).

A caloric quotient of 20.92 kJ was applied in the three energy system calculations (Gastin, 2001). Total energy demand was calculated as the sum of the three energy systems in kJ (Campos et al., 2012). The relative energy system contributions were calculated in % compared to total energy demand.

### Statistical analyses

All parameters were analyzed using GraphPad Prism 9.4.1 (GraphPad Prism Software Inc., La Jolla, CA, USA) and data are presented as mean ± standard deviation (SD). Normality of the data was analyzed using the Shapiro−Wilk test. All physiological variables, energetic contributions, and metabolic flexibility during HICYT were analyzed using a two-way (group x time) repeated-measures analysis of variance (ANOVA) with the Greenhouse-Geisser correction for violation of the sphericity assumption. If the main effect was significant, a Bonferroni post-hoc test was performed to compare among different conditions. The significance level was set at *p* < 0.05. The effect sizes of repeated-measures ANOVA were calculated as partial eta squared [ $\left.\eta_{p}^{2}\right]$ and Cohen’s [*d*] was utilized to indicate between different conditions. Thresholds for small, medium and large effects were considered ≥0.01, ≥0.06, and ≥0.14 for partial eta squared [ $\eta_{p}^{2}$ ] and ≥0.2, ≥0.5, and ≥0.8 for Cohen’s [*d*], respectively (Fritz et al., 2012). Additionally, all data of both groups including pre- and post-tests (n = 40) were analyzed with two-tailed Pearson’s correlation among absolute $\dot{V}$ O_2max_ vs absolute *W*_Oxi_, v $\dot{V}$ O_2max_ (km·h^−1^) vs absolute *W*_Oxi_, absolute $\dot{V}$ O_2max_ vs CHOox, and absolute $\dot{V}$ O_2max_ vs FATox (g·min^−1^).

## Results

### $\dot{V}O_{2\max}\text{ between HIICY and TIHY}$

**FIGURE 3:**
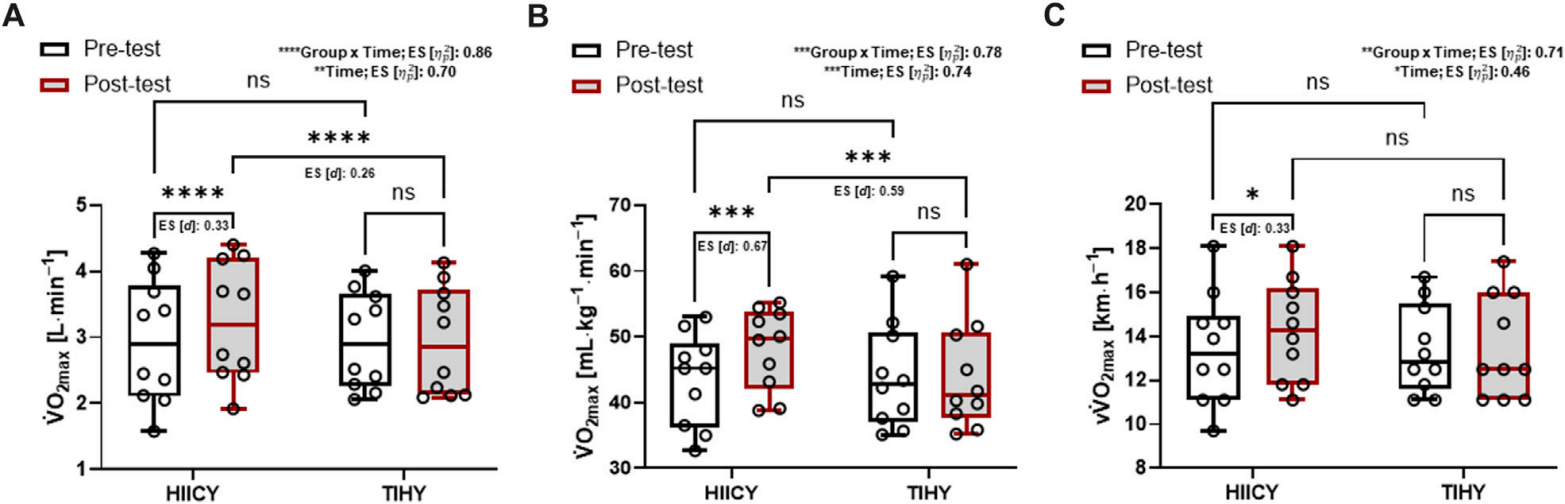
Time and group × time interaction effects and comparisons of absolute and relative $\dot{V}$ O_2max_ and v $\dot{V}$ O_2max_ after 4-week interventions. **(A)** Absolute values of $\dot{V}$ O_2max_ in HIICY and TIHY groups between pre- and post-tests **(B)** relative values of $\dot{V}$ O_2max_ in HIICY and TIHY groups between pre- and post-tests, and **(C)** velocity at $\dot{V}$ O_2max_ in HIICY and TIHY groups between pre- and post-tests. ES; effect sizes, $\dot{V}$ O_2max_; maximal oxygen uptake, v $\dot{V}$ O_2max_; velocity at $\dot{V}$ O_2max_, HIICY; high-intensity interval cardio yoga, TIHY; traditional interval hatha yoga. ns; *p* > 0.05, *p < 0.05, ***p < 0.001, ****p < 0.0001.

Two-way repeated-measures ANOVA for absolute, relative $\dot{V}$ O_2max_ and v $\dot{V}$ O_2max_ indicated significant time and group × time interaction effects (time effect: *p* = 0.0014; [ $\left.\eta_{p}^{2}\right]$ : 0.70, group × time interaction: *p* < 0.0001; [ $\left.\eta_{p}^{2}\right]$ : 0.86, time effect: *p* = 0.0007; [ $\left.\eta_{p}^{2}\right]$ : 0.74, group × time interaction: *p* = 0.0003; [ $\left.\eta_{p}^{2}\right]$ : 0.78, *p* = 0.0224; [ $\left.\eta_{p}^{2}\right]$ : 0.46, group × time interaction: *p* = 0.0011; [ $\left.\eta_{p}^{2}\right]$ : 0.71, respectively) (Figure 3). The absolute and relative $\dot{V}$ O_2max_ values of the HIICY group in the post-test were significantly higher compared with the pre-test and compared with the post-test of the TIHY group (absolute $\dot{V}$ O_2max_: *p* < 0.0001; [*d*]: 0.33, *p* < 0.0001; [*d*]: 0.26, relative $\dot{V}$ O_2max_, *p* = 0.0002; [*d*]: 0.67, *p* = 0.0002; [*d*]: 0.59, respectively) (Figures 3A, B). Furthermore, v $\dot{V}$ O_2max_ of the HIICY group in the post-test was significantly higher than in the pre-test (*p* = 0.0154; [*d*]: 0.33) (Figure 3C).

### Physiological parameters during HICYT

There was no significant difference in anthropometric data between HIICY and TIHY groups. Table 3 shows the physiological parameters during 10-min HICYT pre- and post-tests between different groups. The values of absolute and relative $\dot{V}$ O_2peak_, $\dot{V}$ O_2mean_, HR_peak_, HR_mean_, and RER indicated significant time effects (*p* = 0.0263; effect size [ $\eta_{p}^{2}$ ] : 0.44, *p* = 0.0121; [ $\left.\eta_{p}^{2}\right]$ : 0.52, *p* = 0.0031; [ $\left.\eta_{p}^{2}\right]$ : 0.64, *p* = 0.0053; [ $\left.\eta_{p}^{2}\right]$ : 0.60, *p* = 0.0163; [ $\left.\eta_{p}^{2}\right]$ : 0.50, *p* = 0.0061; [ $\left.\eta_{p}^{2}\right]$ : 0.59, *p* = 0.0013; [ $\left.\eta_{p}^{2}\right]$ : 0.70, respectively). Furthermore, a significant group × time interaction effect for ∆La^−^ (*p* = 0.0198; [ $\eta_{p}^{2}$ ] : 0.47) was observed (Table 3).

**TABLE 3:** Physiological parameters during the 10-min high-intensity cardio yoga test between different groups (HIICY; n = 10 and TIHY; n = 10).

Parameters	HIICY		TIHY		Significance *p* (ES $\eta_{p}^{2}$ )		
	Pre-test	Post-test	Pre-test	Post-test	Group	Time	Group x Time
$\dot{V}$ O_2peak_ [L∙min^−1^]	2.73 ± 0.74	2.90 ± 0.82	2.62 ± 0.54	2.77 ± 0.61	ns	*0.0263 (0.44)	ns
$\dot{V}$ O_2peak_ [mL∙kg^−1^∙min^−1^]	40.97 ± 5.71	43.14 ± 6.37	39.22 ± 6.10	41.60 ± 6.10	ns	*0.0121 (0.52)	ns
$\dot{V}$ O_2mean_ [L∙min^−1^]	2.37 ± 0.63	2.55 ± 0.66	2.29 ± 0.44	2.43 ± 0.51	ns	**0.0031 (0.64)	ns
$\dot{V}$ O_2mean_ [mL∙kg^−1^∙min^−1^]	35.42 ± 4.39	38.00 ± 4.89	34.26 ± 4.76	36.48 ± 5.10	ns	**0.0053 (0.60)	ns
RER [ $\dot{V}$ CO_2_/ $\dot{V}$ O_2_]	1.10 ± 0.05	1.15 ± 0.04	1.10 ± 0.03	1.10 ± 0.06	ns	*0.0163 (0.50)	ns
HR_peak_ [beats∙min^−1^]	188.70 ± 5.96	183.80 ± 8.46	182.70 ± 7.69	180.80 ± 7.58	ns	**0.0061 (0.59)	ns
HR_mean_ [beats∙min^−1^]	177.90 ± 5.60	171.70 ± 8.49	173.80 ± 8.30	168.3 ± 8.12	ns	**0.0013 (0.70)	ns
ΔLa^−^ [mmol∙L^−1^]	8.57 ± 1.54	7.62 ± 2.98	7.44 ± 2.90	8.51 ± 1.65	ns	ns	*0.0198 (0.47)
La^−^_peak_ [mmol∙L^−1^]	9.48 ± 1.58	8.73 ± 2.98	8.83 ± 2.90	9.59 ± 1.63	ns	ns	ns

Data shown as mean ± standard deviation (n = 20). HIICY, high-intensity interval cardio yoga; TIHY, traditional interval hatha yoga; ES, effect size; $\dot{V}$ O_2peak_, peak oxygen uptake; $\dot{V}$ O_2mean_, mean oxygen uptake; RER, respiratory exchange ratio; HR_peak_, peak heart rate; HR_mean_, mean heart rate; ∆La^−^, delta lactate; La^−^_peak_, peak lactate; ns, *p* > 0.05, *p < 0.05, **p < 0.01.

### Energetic contribution during HICYT

**FIGURE 4:**
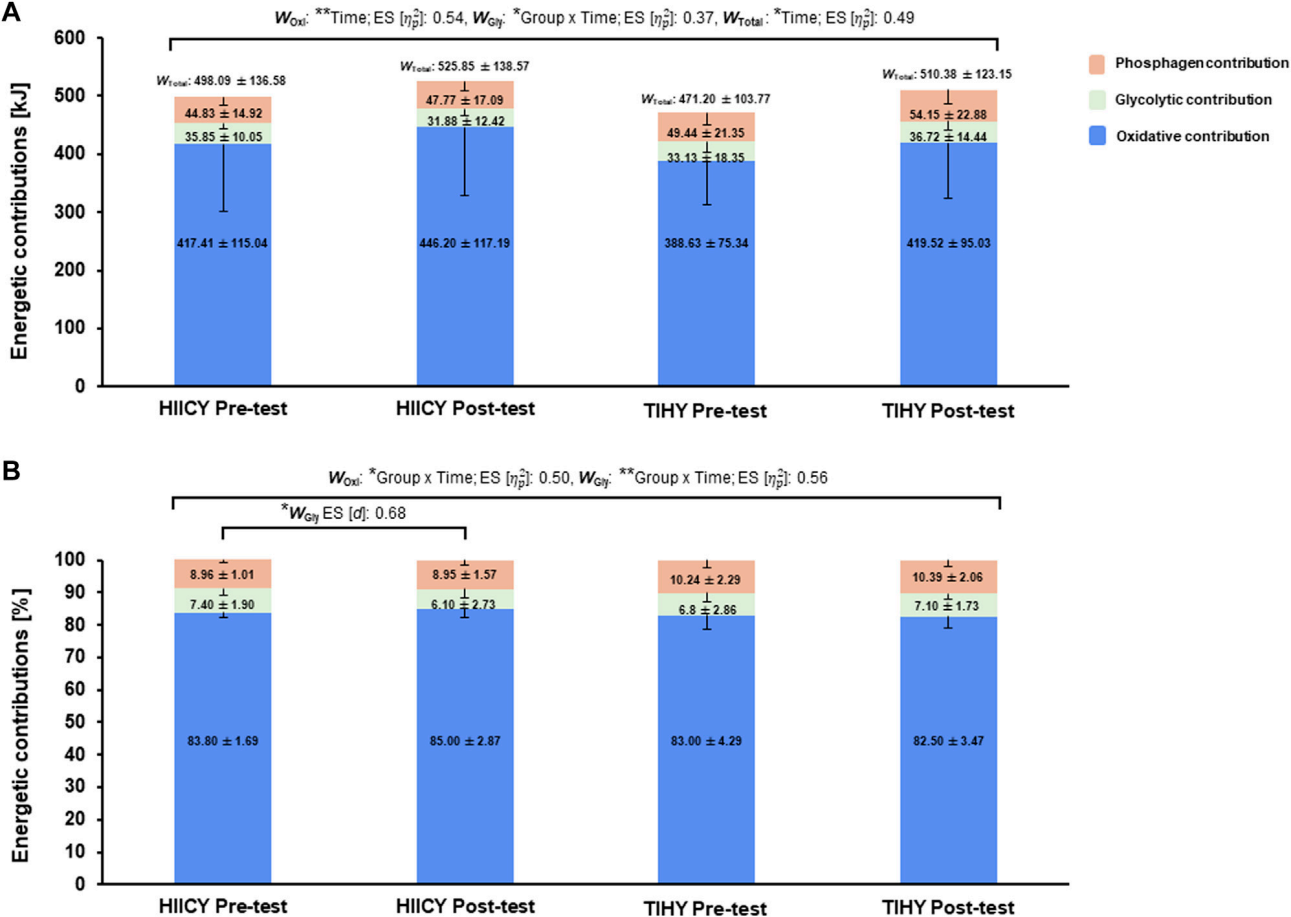
Time and group × time interaction effects and comparisons of energetic contributions during the HICYT after 4-week interventions. **(A)** Comparisons of three energy contributions in kJ between pre- and post-test in HIICY and TIHY groups, respectively and **(B)** comparisons of three energy contributions in % between pre- and post-testing HIICY and TIHY groups, respectively. ES; effect sizes, *W*_Oxi_; oxidative energy demand, *W*_Gly_; glycolytic energy demand, *W*_Total_; total energy demand, HICYT; high-intensity cardio yoga test, HIICY; high-intensity interval cardio yoga, TIHY; traditional interval hatha yoga. *p < 0.05, **p < 0.01.

Two-way repeated-measures ANOVA showed significant time effects in absolute (kJ) for *W*_Oxi_ and *W*_Total_, and a group × time interaction effect in absolute *W*_Gly_ (time effects: *p* < 0.01; [ $\left.\eta_{p}^{2}\right]$ : 0.54, *p* = 0.0173; [ $\left.\eta_{p}^{2}\right]$ : 0.49, group × time interaction: *p* = 0.0479; [ $\left.\eta_{p}^{2}\right]$ : 0.37, respectively). Moreover, significant group × time interaction effects in relative (%) *W*_Oxi_ and *W*_Gly_ were observed (*p* = 0.014; [ $\left.\eta_{p}^{2}\right]$ : 0.50, *p* = 0.0084; [ $\left.\eta_{p}^{2}\right]$ : 0.56, respectively) (Figures 4A, B). The relative *W*_Gly_ value of the HIICY group in the post-test was significantly lower than in the pre-test (*p* = 0.0376; [*d*]: 0.68) (Figure 4B).

### Metabolic flexibility including FATox and CHOox during HICYT

**FIGURE 5:**
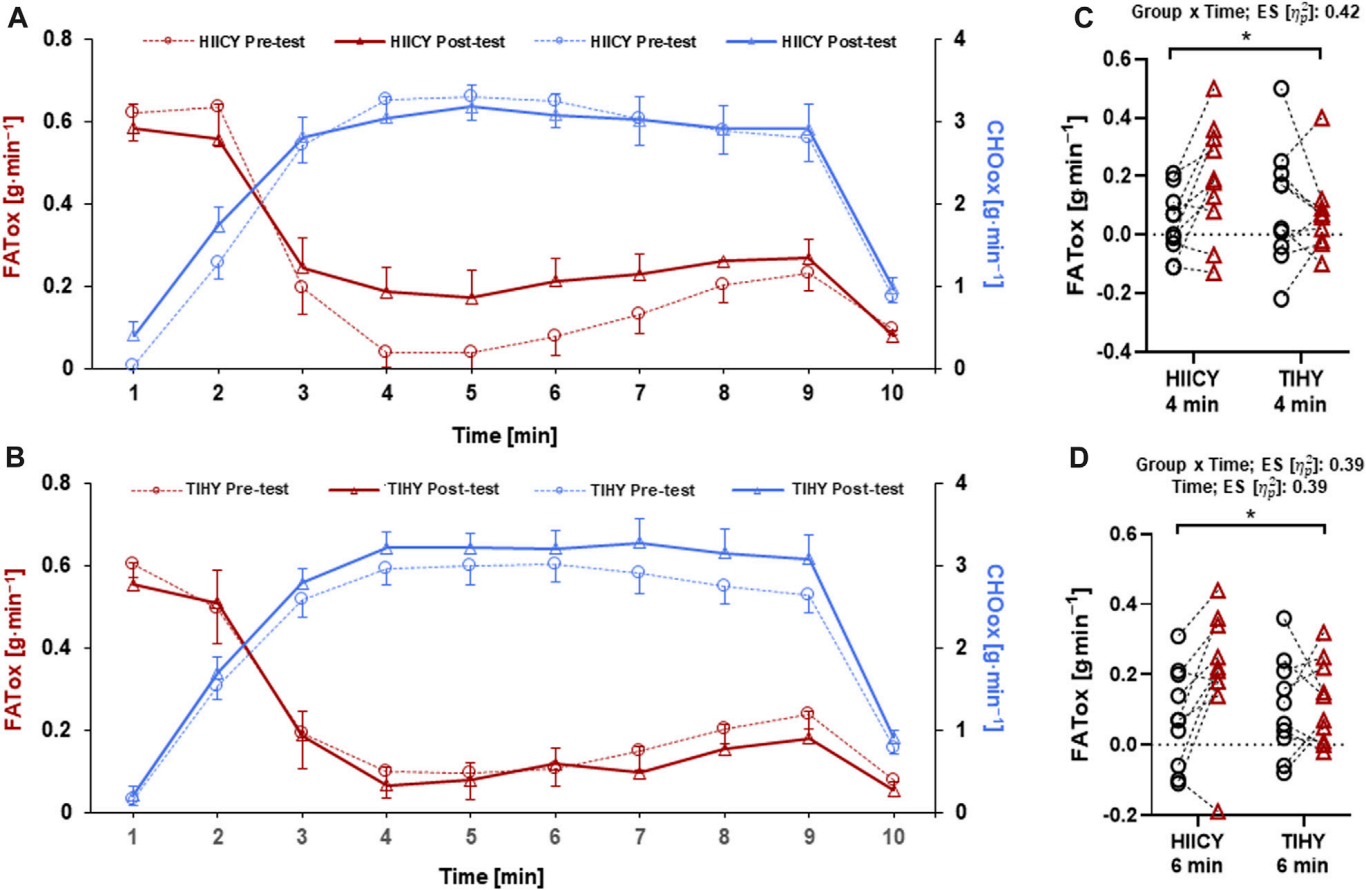
FATox and CHOox between HIICY and TIHY during the HICYT. **(A)** FATox and CHOox in the HIICY group between pre- and post-testing, **(B)** FATox and CHOox in the TIHY group between pre- and post-testing, **(C)** group × time interaction effect of FATox at 4 min during the HICYT, and **(D)** time and group × time interaction effects of FATox at 6 min during the HICYT. FATox; fat oxidation, CHOox; carbohydrate oxidation, HIICY; high-intensity interval cardio yoga, TIHY; traditional interval hatha yoga, HICYT; high-intensity cardio yoga test. *p < 0.05.

Two-way repeated-measures ANOVA showed group × time interaction effects at the fourth and time and group × time interaction effects at sixth minute of FATox (*p* = 0.0312; effect size [ $\left.\eta_{p}^{2}\right]$ : 0.42, *p* = 0.0412; [ $\left.\eta_{p}^{2}\right]$ : 0.39, *p* = 0.0386; [ $\left.\eta_{p}^{2}\right]$ : 0.39, respectively) (Figures 5A–D). Otherwise, no significant main effect of FATox and CHOox and between pre- and post-tests were observed.

### Relationships between metabolic flexibility, energetic contributions and physiological variables

**FIGURE 6:**
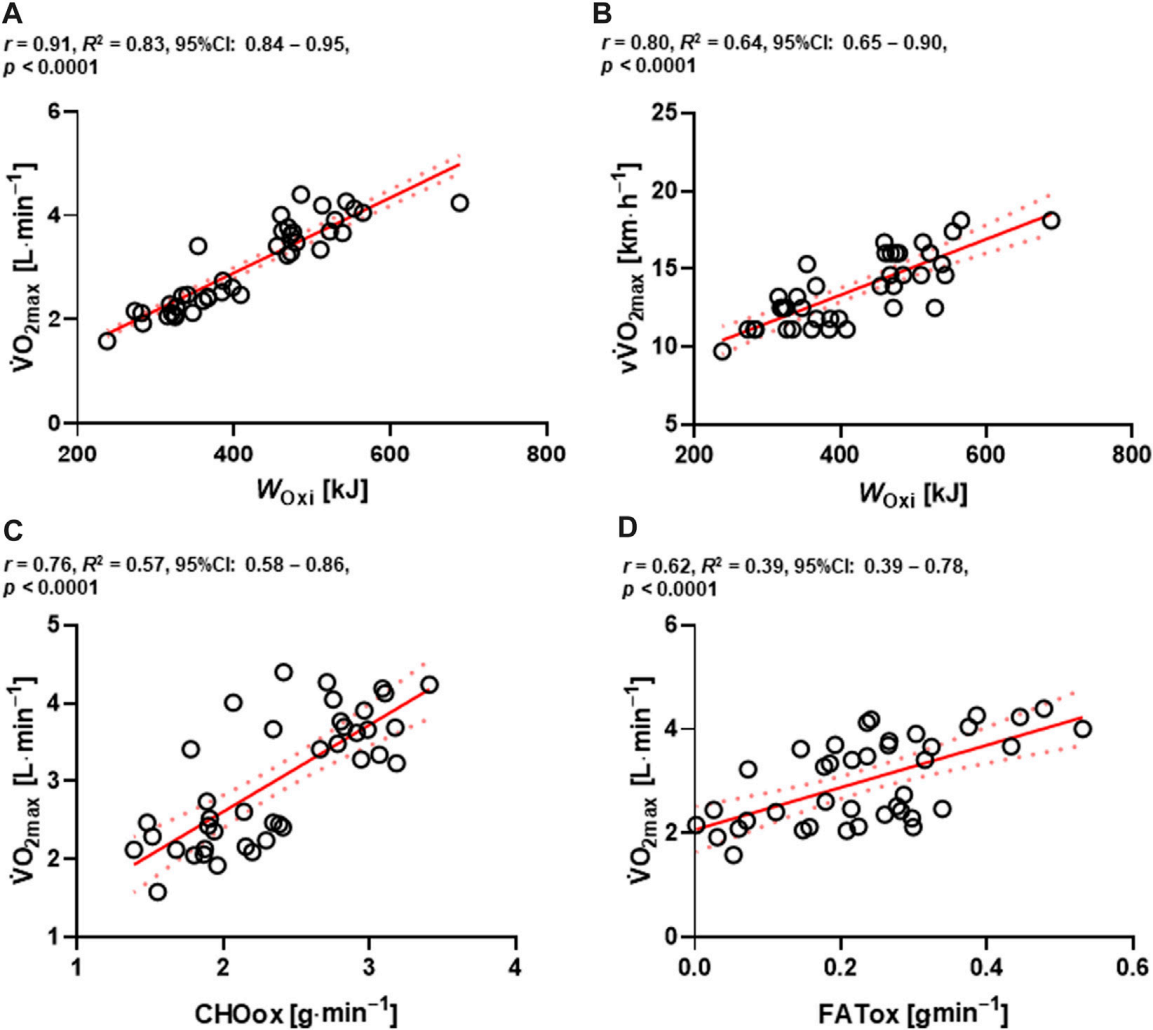
Relationships between absolute $\dot{V}$ O_2max_, v $\dot{V}$ O_2max_ and absolute *W*_Oxi_, and CHOox, FATox and absolute $\dot{V}$ O_2max_. **(A)** two-tailed Pearson’s correlation between absolute *W*_Oxi_ and absolute $\dot{V}$ O_2max_, **(B)** two-tailed Pearson’s correlation between absolute *W*_Oxi_ and v $\dot{V}$ O_2max_, **(C)** two-tailed Pearson’s correlation between CHOox and absolute $\dot{V}$ O_2max_, and **(D)** two-tailed Pearson’s correlation between FATox and absolute $\dot{V}$ O_2max_. CI; confidence interval, $\dot{V}$ O_2max_; maximal oxygen uptake, *W*_Oxi_; oxidative energy demand, v $\dot{V}$ O_2max_; velocity at $\dot{V}$ O_2max_, CHOox; carbohydrate oxidation, FATox, fat oxidation. ****p < 0.0001.

High positive correlations were found between *W*_Oxi_ in kJ, and absolute $\dot{V}$ O_2max_, as well as v $\dot{V}$ O_2max_ (*W*_Oxi_ vs absolute $\dot{V}$ O_2max_: *r* = 0.91, *R* ^*2*^ = 0.83, 95%CI: 0.84–0.95; *p* < 0.0001, *W*_Oxi_ vs v $\dot{V}$ O_2max_: *r* = 0.80, *R* ^*2*^ = 0.64, 95%CI: 0.65–0.90; *p* < 0.0001, respectively) (Figures 6A, B). Furthermore, a high positive correlation between CHOox and absolute value of $\dot{V}$ O_2max_ (*r* = 0.76, *R* ^*2*^ = 0.57, 95%CI: 0.58–0.86; *p* < 0.0001) and moderate positive correlation between FATox and absolute value of $\dot{V}$ O_2max_ (*r* = 0.62; *R* ^*2*^ = 0.39; 95%CI: 0.39–0.78; *p* < 0.0001) were found (Figures 6C, D).

## Discussion

Training effects of HIIT and HY on health, oxidative capacity, and metabolism have been well reported previously. To date, however, there was no interventional approach to HIICY. It is unclear whether HIICY as HIHY can improve cardiometabolic fitness parameters such as $\dot{V}$ O_2max_, energetic contributions, and metabolic flexibility in physically active adults. To the best of our knowledge, this is the first study to investigate how different intensities during 4-week tempo-based HY practices (HIICY and TIHY) affected $\dot{V}$ O_2max_, energetic contributions, and metabolic flexibility. Our major findings indicate that time and group × time interaction effects in absolute and relative $\dot{V}$ O_2max_ after 4-week HIICY and TIHY training regimens were observed. $\dot{V}$ O_2max_ was improved between pre- and post-testing in the HIICY but not in TIHY. During the HICYT, time and group × time interaction effects among *W*_Oxi_ in kJ, *W*_Total_, *W*_Gly_ in kJ, *W*_Oxi_ in %, and *W*_Gly_ in % were found. The value of *W*_Gly_ in % was decreased only between pre- and post-tests in the HIICY group. Furthermore, time and group × time interaction effects in FATox showed at the fourth and sixth minute after HIICY and TIHY, and moderate to high correlations among $\dot{V}$ O_2max_ vs *W*_Oxi_, v $\dot{V}$ O_2max_ vs *W*_Oxi_, $\dot{V}$ O_2max_ vs CHOox, and $\dot{V}$ O_2max_ vs FATox were found.

Regarding maximal aerobic performance, 4-week HIICY improved $\dot{V}$ O_2max_ and v $\dot{V}$ O_2max_ while 10-min TIHY showed no increase in $\dot{V}$ O_2max_ between pre- and post-tests in physically active individuals. In this regard, at least 10-min of Surya Namaskar B (SS) compared with general HY achieved an intensity that could improve cardiometabolic fitness (Hagins et al., 2007). Furthermore, physiological profiling of Surya Namaskar B between 3 s and 12 s tempos indicated exercise intensities that were moderate-to high-intensity and low-intensity (Potiaumpai et al., 2016). A recent study by Lee et al. (2021) showed that Surya Namaskar B at a fast tempo of 1.5 s was suitable for HIIT (>90% of HR_max_, ≥6 METs; vigorous/heavy, >4 mmol·L^−1^ La^−^; zone 3). These outcomes suggest that fast tempo-based HY practice is necessary, if the goal is to increase endurance performance and cardiometabolic fitness. The adjustment of exercise intensity and duration between work and recovery intervals alters the relative demand for specific metabolic pathways within muscle cells, oxygen delivery to the working muscle, and subsequent adaptations. These changes occur at the cellular and systemic levels depending on the specific nature of the training program (Laursen and Jenkins, 2002; Sandbakk et al., 2013; Franchini et al., 2019). HIIT with long-duration (5–10 min) and shorter intervals of higher intensity such as seen in supramaximal intensity have been shown to be an effective metabolic stimulus for improving $\dot{V}$ O_2max_. Long-duration intervals such as 5–10 min increase $\dot{V}$ O_2max_ and $\dot{V}$ O_2_ at ventilatory thresholds in national-level junior cross-country skiers (Sandbakk et al., 2013). Therefore, previous study outcomes support our results showing that the long-duration during HIIT is a relevant factor in increasing $\dot{V}$ O_2max_ in well-trained athletes as well as in the general population (Gaskill et al., 1999; Billat, 2001; Laursen and Jenkins, 2002; Seiler, 2010; Seiler et al., 2013).

HR_peak_ and HR_mean_ tended to improve post-testing in the HIICY group. Significant increases in pulmonary oxygen uptake and skeletal muscle oxygen demand cause HIIT-induced cardiovascular adaptations during intensive and prolonged HIIT sessions. Exercise-induced improvement in $\dot{V}$ O_2max_ is associated with enlarged red blood cell volume, which leads to greater oxygen-rich blood capacity and increased stroke volume. Thus, it can affect an increase in O_2_ transport capacity (Predel, 2014). Sabag et al. (2022) indicated in their topical review that systolic and diastolic function, maximum cardiac output, capillary density, and stroke volume were increased after HIIT. Indeed, the usefulness of HIIT in stimulating significant improvements in cardiometabolic fitness, left ventricular ejection fraction, and pathological left ventricular remodeling of participants was emphasized for those who completed 36 sessions of HIIT over 2–3 months (Hsu et al., 2019). These preliminary findings suggest that increased stroke volume can deliver more oxygen per heartbeat (Kohn et al., 2011). A reduced HR at submaximal intensity after HIIT may be one of the major HIIT-induced physiological adaptations, and one which also associates with increased $\dot{V}$ O_2max_ (Acevedo and Goldfarb, 1989; Kubukeli et al., 2002).

The reduced value of %*W*_Gly_ with increased $\dot{V}$ O_2max_ between pre- and post-testing in the HIICY group (Figure 4B) is likely associated with increased HIIT-induced metabolic responses (Batacan et al., 2017; MacInnis and Gibala, 2017; Chrøis et al., 2020; Sabag et al., 2022). Generally, cellular stress is proportional to exercise intensity and there is strong evidence that higher exercise intensities induce elevated molecular responses compared to moderate intensities (Egan and Zierath, 2013). This may be because higher rates of fuel utilization relies more on carbohydrate oxidation, uses more glycogen, and increases ATP turnover (Howlett et al., 1998; Van loon et al., 2001). Consequently, it activates signaling pathways involved in mitochondrial biogenesis following intracellular lactate production, creatine kinase, ADP, and AMP accumulation (Howlett et al., 1998; Van loon et al., 2001) phosphorylation of AMP-activated protein kinase, p38 mitogen-activated protein kinase, and Ca^2+^/calmodulin-dependent protein kinase II, and expression of peroxisome proliferator-activated receptor gamma coactivator 1-alpha (PGC-1α) mRNA (Gibala et al., 2009; Egan et al., 2010; Little et al., 2011; Kristensen et al., 2015; Metcalfe et al., 2015; Bersiner et al., 2023). Regular and repeated activation of these pathways increases mitochondrial density (Coffey and Hawley, 2007). Greater activation of these specific kinases were induced by high-intensity exercise compared to low-intensity exercise resulting in greater expression of mRNA for PGC-1α which is a master regulator of mitochondrial biogenesis (Egan et al., 2010). PGC-1α is responsible for the activation of mitochondrial transcription factors such as nuclear respiratory factors 1 and 2 and the mitochondrial transcription factor A (Knuiman et al., 2015). Downstream of these metabolic signals, mitochondrial protein synthesis has been shown to result in higher mitochondrial biogenesis in response to sustained training performed at higher intensities with a given amount of exercise (Di Donato et al., 2014).

Furthermore, numerous studies have shown an increased density of monocarboxylate transporters (MCT) 1 and 4 after HIIT (Burgomaster et al., 2007; Perry et al., 2008; McGinley and Bishop, 2016). Higher MCT 1 and 4 density increased lactate transport and likely supported a reduction in glycogen breakdown and La^−^ at a given intensity (Perry et al., 2008). Accordingly, group × time interaction effect of energetic contributions was observed and the relative value of *W*_Gly_ post-testing in the HIICY group was decreased (Figure 4B). Because of the aforementioned physiological adaptations, oxidative capacity might be improved, which would in turn increase the lactate elimination rate and increase ATP re-synthesis during high-intensity aerobic exercise (Burgomaster et al., 2008; MacInnis and Gibala, 2017; Brooks, 2018; Hwang et al., 2022; Yang et al., 2023). Indeed, muscle glycogen is likely conserved, delaying the onset of muscle fatigue and improving oxidative exercise performance (Hearris et al., 2018). Also, high positive correlations among *W*_Oxi_ in kJ and absolute $\dot{V}$ O_2max_, as well as v $\dot{V}$ O_2max_ in our study outcomes, support the conclusion of a HIIT-induced physiological adaptation (Figure 6).

In terms of metabolic flexibility, time and group × time interaction effects of FATox at the fourth and sixth during the HICYT between HIICY and TIHY groups and moderate to high correlations among $\dot{V}$ O_2max_ vs CHOox, and $\dot{V}$ O_2max_ vs FATox were found (Figures 5, 6). These results in FATox may be affected by peripheral improvements at the level of muscle cells, such as in mitochondrial function. Such changes have been, reported in a number of earlier studies that have detailed improvements in mitochondrial function and insulin sensitivity, and which together represent improved metabolic flexibility (Sandbakk et al., 2013; Chrøis et al., 2020; Yang et al., 2022a; Sabag et al., 2022). Furthermore, a higher percentage of FATox during HIIT than short-interval training in about half of previous studies was observed (Astorino and Schubert, 2018). In detail, significant increases in *β*-hydroxyacyl acyl-CoA dehydrogenase, citrate synthase, fatty acid-binding protein, carnitine palmitoyl transferase I or fatty acid translocase/cluster of differentiation 36, and expression of PGC-1α are likely responsible for increased FATox after HIIT (Tunstall et al., 2002; Perry et al., 2008; Talanian et al., 2010; Astorino and Schubert, 2018; Warren et al., 2020). Burgomaster et al. (2008) reported that 18 sessions of HIIT for 6 weeks increased glucose transporter isoform 4 content and promoted glucose uptake during recovery, and greater muscle glycogen levels. Several weeks of HIIT can increase muscle FATox capacity which is associated with more hydroxyacyl-CoA dehydrogenase activity and improved insulin resistance. Also of note, HY has been shown to reduce adipose cell concentrations in the visceral area, diminishing or minimizing the excess free fatty acids released by adipose cells (Khoshnaw and Ghadge, 2021).

In sum, this study has demonstrated increased cardiometabolic fitness, including $\dot{V}$ O_2max_, energetic contributions, and metabolic flexibility after a 30-min HIICY regimen (2 sets, 3 times per week) for 4 weeks compared with TIHY in physically active adults. Moreover, we investigated whether HIICY for at least 4 weeks significantly affected the cardiometabolic biomarkers described above. Therefore, we suggest that the positively affected outcomes likely depend on exercise intensity influencing the activation of PGC-1α, the master regulator of mitochondrial biogenesis in human skeletal muscle (Egan et al., 2010; Gibala et al., 2012). Also, the high-intensity level should be maintained as much as possible during repeated sessions, with this being more important than maintaining duration or frequency to induce an increase in $\dot{V}$ O_2max_ (Hickson and Rosenkoetter, 1981). Therefore, HIICY consisting of a specific sequence of 19 asanas is a fast tempo-based approach, which can provide cardiometabolic health benefits in physically active individuals.

This study does have some limitations. First, it only targeted one cohort of physically active adults and the HIICY approach should be investigated in more diverse populations such as in athletes, sedentary people, and clinical populations in further studies. Second, the small sample size might have influenced our results. Therefore, more participants should be recruited in a future study. Third, experimenting with efficient periodization to progressively increase HY intensity from TIHY to HIICY should be considered. Finally, more direct measurements during training using proteomics and metabolomics, and the measurement of fluorescent protein tools should be investigated to determine molecular responses in the future.

## Conclusion

Our findings indicate that 4-week of HIICY compared with TIHY improved cardiometabolic fitness, oxidative capacity, and metabolic flexibility in physically active individuals. Therefore, HIICY is a suitable training regimen for HY-specific HIIT, and is appropriate for relatively healthy individuals who may have HY experience but need time-efficient exercise options. Through our study outcomes, it is once again confirmed that exercise intensity during HY is important to improving $\dot{V}$ O_2max_. While HY is an effective practice in many respects, significant and efficient improvements to cardiometabolic fitness require fast-tempo HIICY. Moreover, a proportionately higher $\dot{V}$ O_2max_ strongly signals greater cardiovascular health and life expectancy at any age. Thus, this substitute system of practice may prevent cardiac/metabolic diseases in the general population. However, HIICY has a very high intensity for exercise beginners. Therefore, it is recommended to start practicing with 3 s tempo-based TIHY during an initial period within the typical linear periodization model for an aerobic base, and then gradually increase the intensity to HIICY.

## Data Availability

The original contributions presented in the study are included in the article/Supplementary material, further inquiries can be directed to the corresponding author.
